# Correction: Impacts of land use on soil carbon, nitrogen, and phosphorus in the Eastern Qilian Mountains

**DOI:** 10.1371/journal.pone.0353942

**Published:** 2026-07-14

**Authors:** Shizhen Xu, Chunli Wang, Junju Zhou, Haihua Shan, Bingxing Li, Wei Shi, Dongxia Zhang, Guofeng Zhu, Xuemei Yang, Wei Wei, Haiyan Ma

The Funding statement for this article is incorrect. The correct Funding statement is as follows: This research was supported by the National Natural Science Foundation of China under grants (42361005) awarded to JJZ, (41867030) awarded to GFZ, (32060373) awarded to XMY, and the Gansu Province Water Conservancy Scientific Experiment Research and Technology Promotion Project (Grant No. 23GSLK086) awarded to HYM. No additional external funding was received for this study.
