# Supplementary material for: Impacts of land use on soil carbon, nitrogen, and phosphorus in the Eastern Qilian Mountains
Source: PLoS One. 2025 Jul 14;20(7):e0326316. doi: 10.1371/journal.pone.0326316 (PMC12258590; doi:10.1371/journal.pone.0326316)
Supplement: S2 File — (DOCX) [file pone.0326316.s002.docx]

| _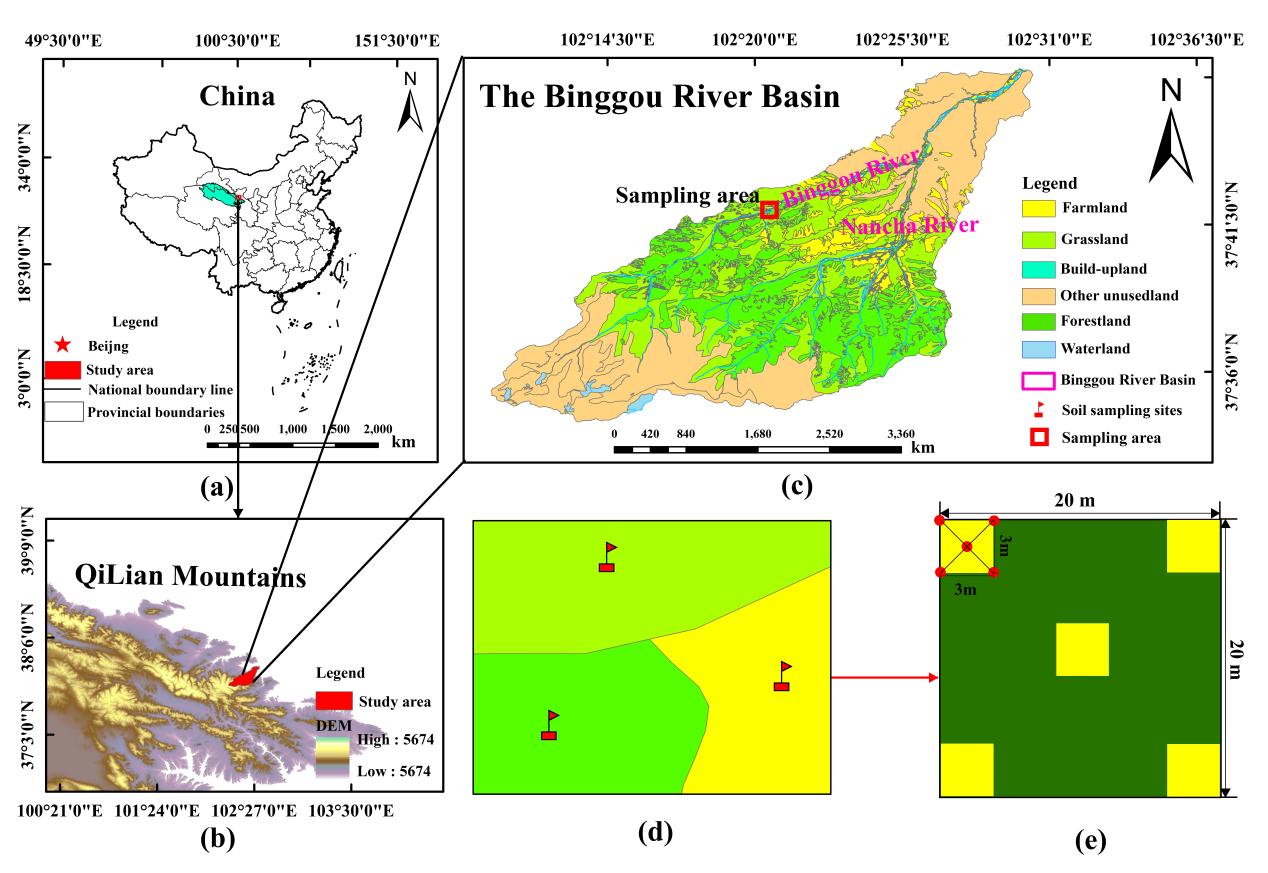_ |
| --- |
| S1 Fig. The sampling location and process of the Binggou River Basin on the Qilian Mountains. |
| 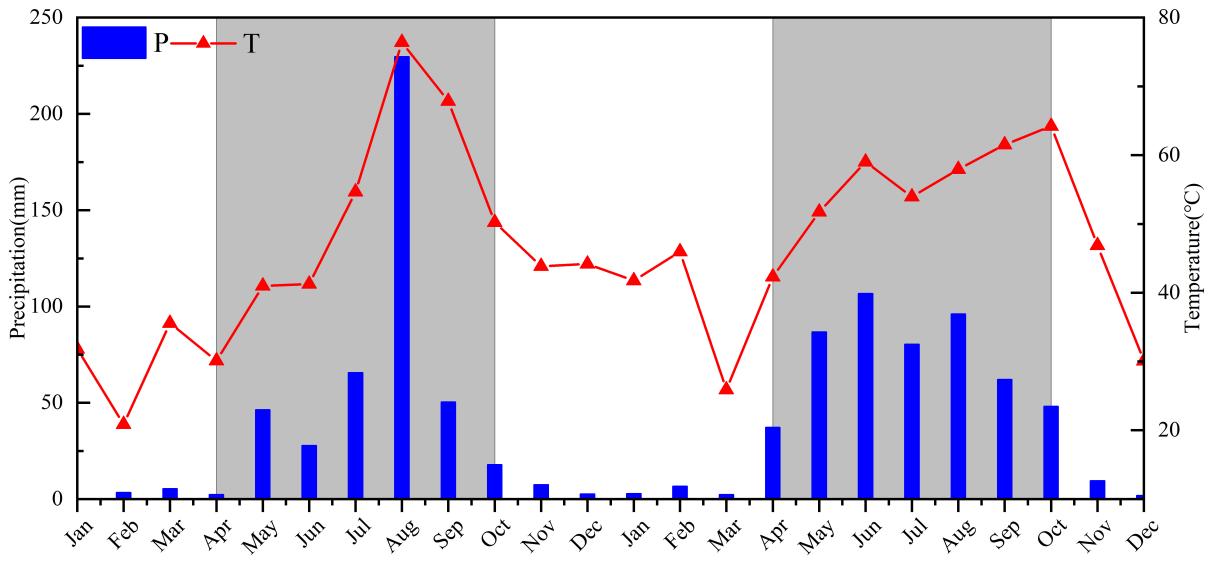 |
| S2 Fig. Distribution of temperature and precipitation in the study area (2018-2019) |
| 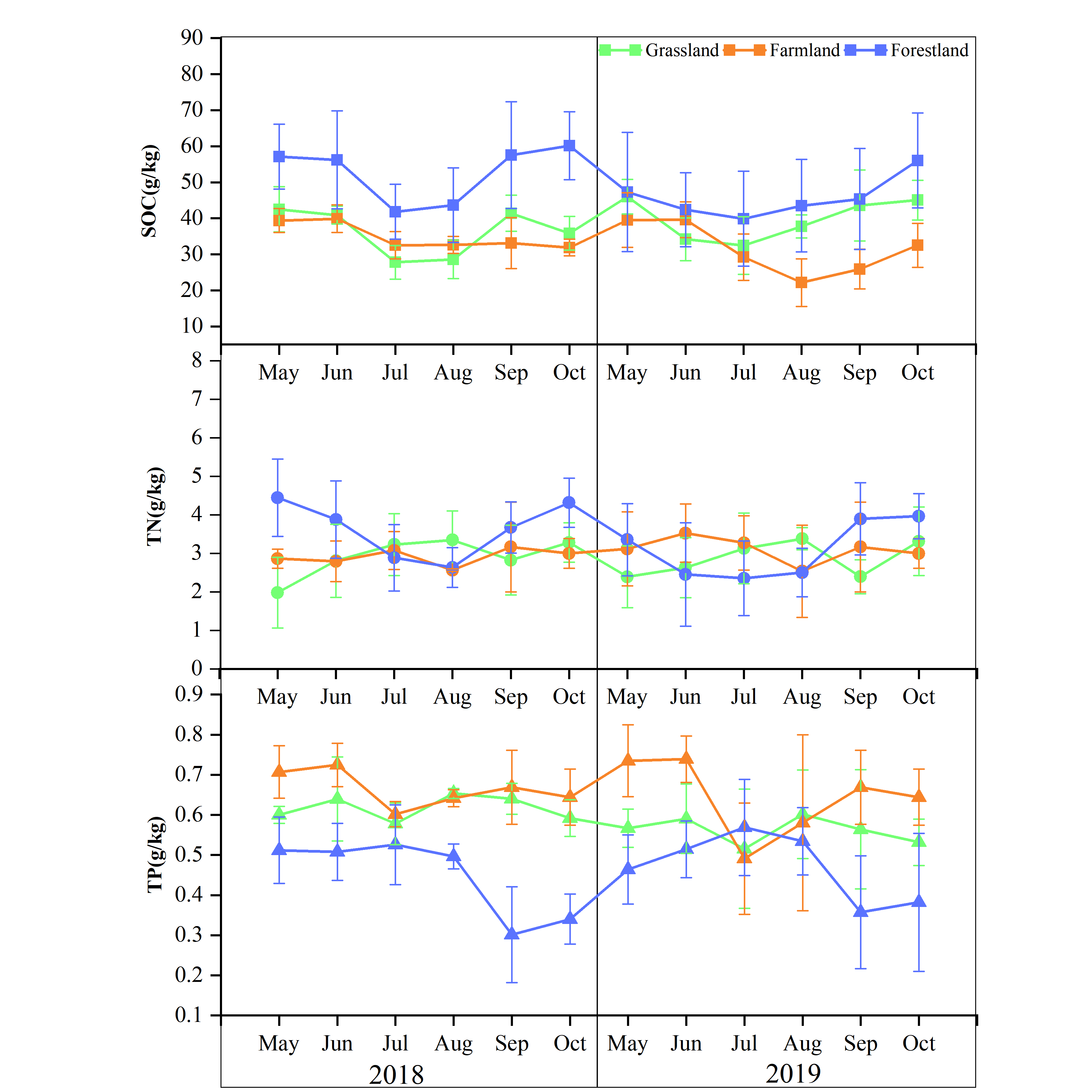 |
| S3 Fig. Concentrations of C, N, and P in time change |
| 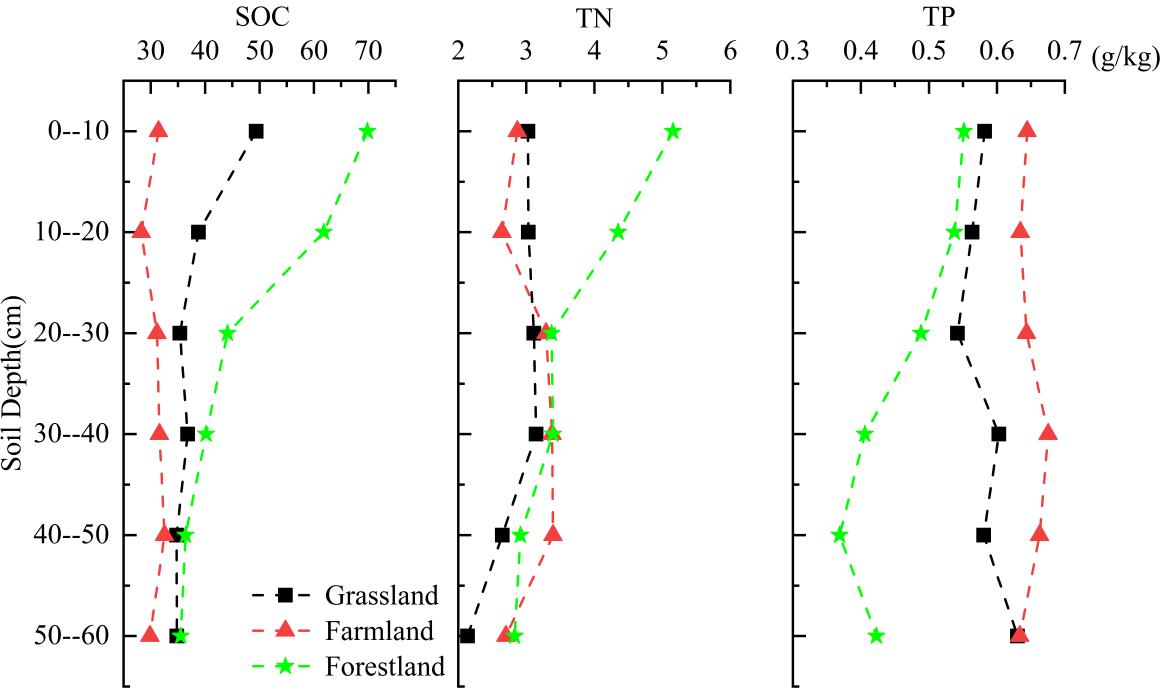 |
| S4 Fig. Concentrations of SOC, TN, and TP in vertical feature |
| 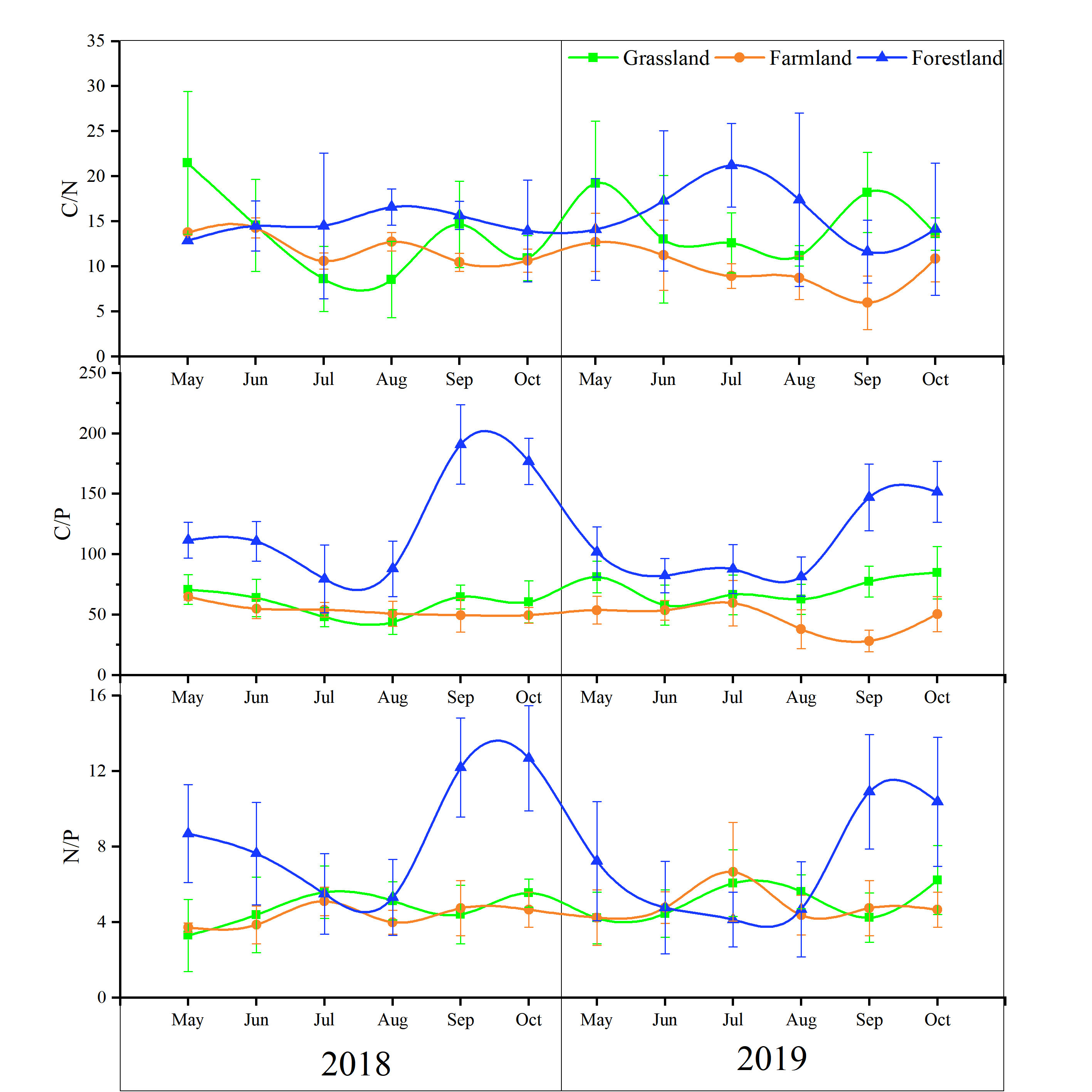 |
| S5 Fig. The ecological stoichiometry of soil in time feature |
| 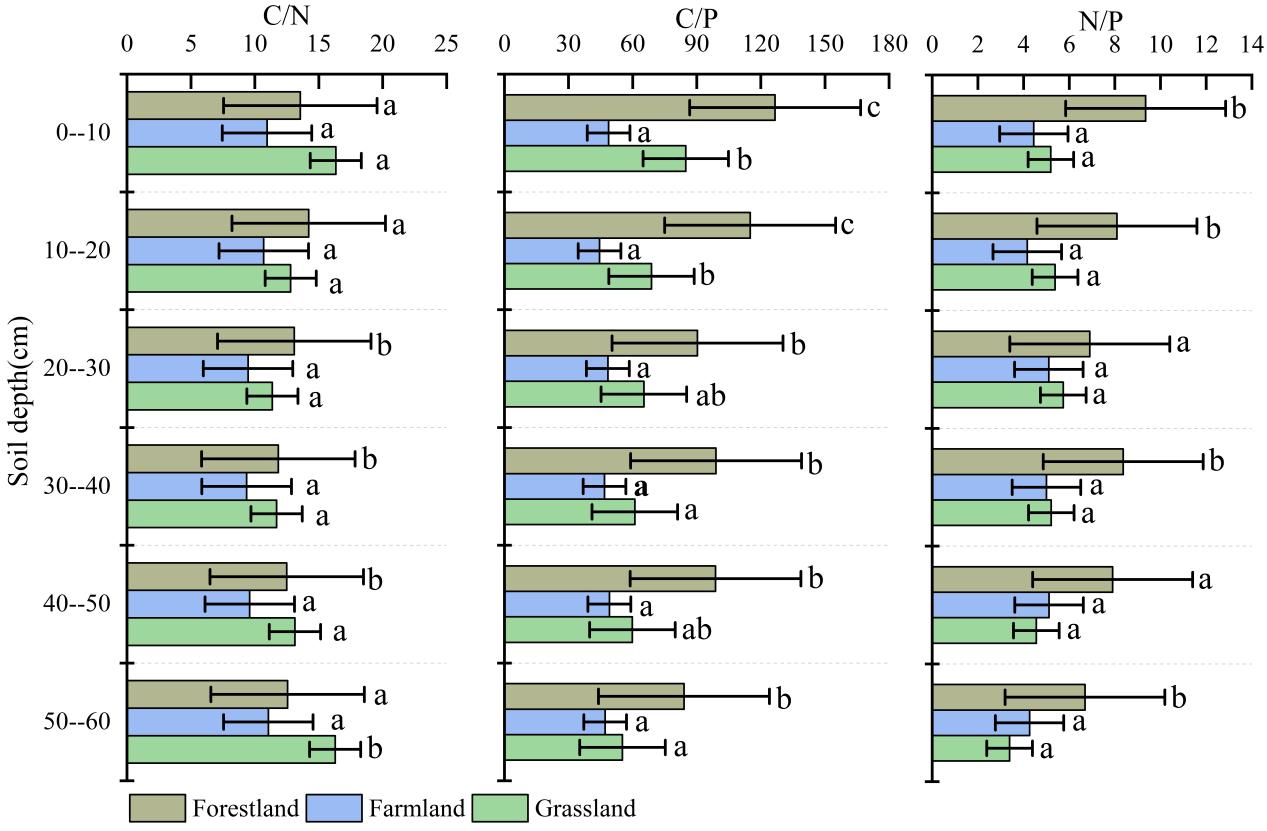 |
| S6 Fig. The ecological stoichiometry of soil in vertical feature.(Different small letters mean a significant difference at the 0.05 level) |
| 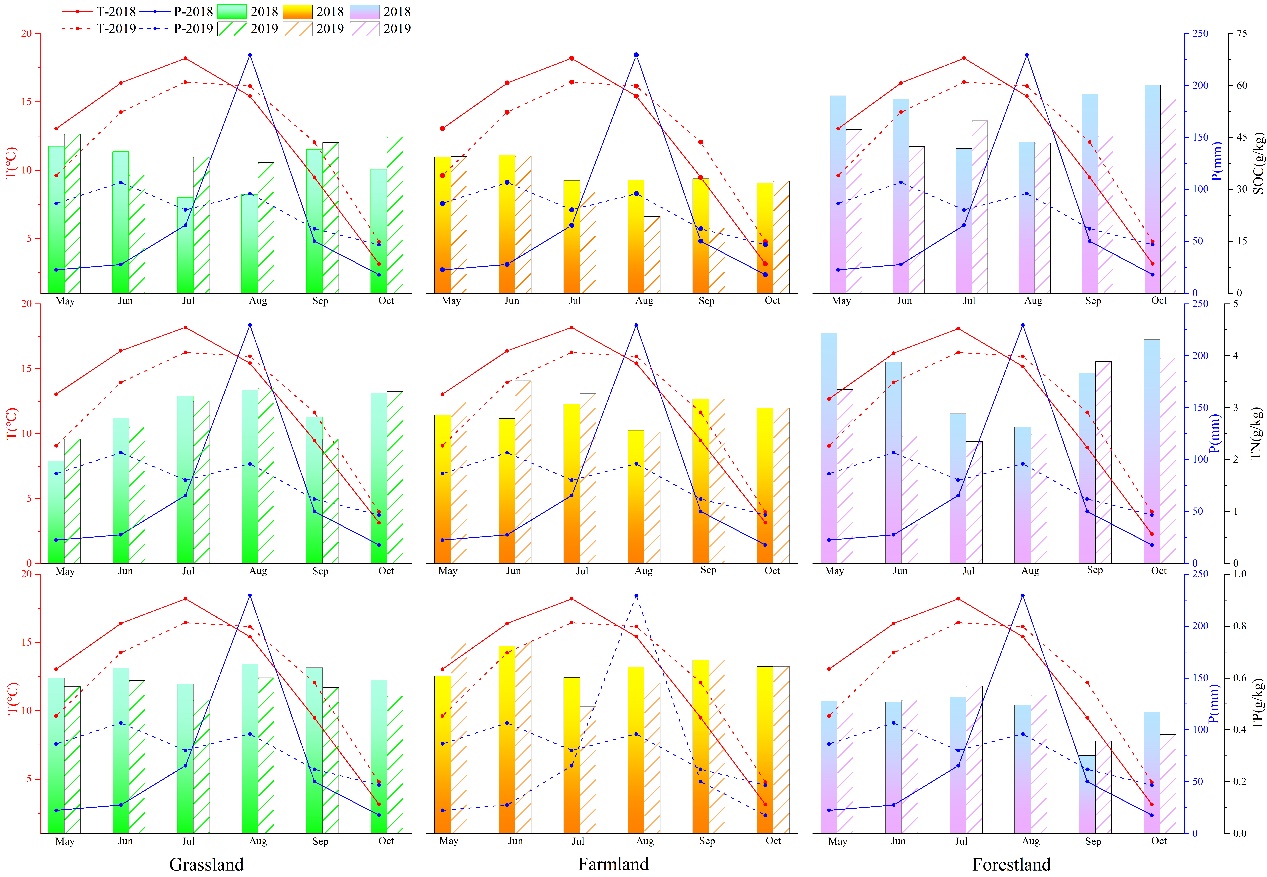 |
| S7 Fig. The relationship between temperature, precipitation and SOC, TN and TP contents in different land use types |
| \|  \| SOC(g/kg) \| TN(g/kg) \| TP(g/kg) \| C/N \| C/P \| N/P \| \| --- \| --- \| --- \| --- \| --- \| --- \| --- \| \| Forestland \| 48.82±7.59 \| 3.52±0.77 \| 0.46±0.09 \| 13.87±6.11 \| 106.13±48.82 \| 7.65±3.22 \| \| Grassland \| 38.32±5.87 \| 2.85±0.44 \| 0.58±0.04 \| 13.45±6.54 \| 66.07±11.80 \| 4.91±0.98 \| \| Farmland \| 30.8±5.94 \| 3.04±0.25 \| 0.65±0.07 \| 10.13±1.98 \| 47.38±8.32 \| 4.67±0.79 \| |
| S1 Table. Soil carbon, nitrogen and phosphorus contents and ecological stoichiometric ratios of different land use types. Values are average±standard error. |
