# Supplementary material for: Impacts of land use on soil carbon, nitrogen, and phosphorus in the Eastern Qilian Mountains
Source: PLoS One. 2025 Jul 14;20(7):e0326316. doi: 10.1371/journal.pone.0326316 (PMC12258590; doi:10.1371/journal.pone.0326316)
Supplement: Attachment — Submitted filename: Minor revision comments.pdf [file pone.0326316.s004.pdf]

Minor revision comments on the manuscript (PONE-D-24-59609R1)

This version of the manuscript responds to the comments of the three reviewers one by one, and the quality of the manuscript has been greatly improved, but more specific and comprehensive information needs to be provided in some details through minor revision. My main suggestions are as follows:

Firstly, in the section “2.1 Description of the Study Area”, It is suggested to supplement the land use and cover changes of the whole study area over the past 10 years, especially the plant composition and community structure characteristics of each sampling site and the tillage of agricultural land.

Secondly, It is proposed to supplement the hydrological connectivity status of the Binggou River Basin, including the interaction of surface runoff in its adjacent catchments.

Thirdly, Please state what damage has been done to the ecological environment in the study area and to what extent.

Others, (1) There is room for improvement in some series of results analysis, for example, in Fig 7, Columns filled with patterns and colors only indicate the meaning of grassland, farmland and forestland are not marked. (2) In terms of the content arrangement of this manuscript, the results and the discussion are separate. It is suggested that the discussion expressions quoting references in the results should be included in the discussion chapter. (3) The evidence (references) cited in the discussion section should have similar research premises to this study, please check. (4) When it comes to the significance level of the impact/difference, it is recommended to indicate a probability value, such as p < xxx.
