# Supplementary material for: Impacts of land use on soil carbon, nitrogen, and phosphorus in the Eastern Qilian Mountains
Source: PLoS One. 2025 Jul 14;20(7):e0326316. doi: 10.1371/journal.pone.0326316 (PMC12258590; doi:10.1371/journal.pone.0326316)
Supplement: Attachment — Submitted filename: Response to Reviewers.doc [file pone.0326316.s003.doc]

Dear Editor and Reviewers:
We are grateful for your consideration of our manuscript entitled "Impacts of Land Use on Soil Carbon, Nitrogen, and Phosphorus Cycling in the Eastern Qilian Mountains" [PONE-D-24-59609], and we also very much appreciate your constructive comments and useful suggestions, which have enabled us to improve the manuscript. All the comments we received on this study have been considered, and we present our reply to each of them separately. We hope the revised manuscript will satisfy you.The following is an illustration of the different coloured revisions in the manuscript with markings: blue with strikethrough are markings removed from the original text, yellow highlights are reworked additions, and red headings are reworked sections.

Reviewer #1: General Comments

This study investigates the impact of land use on the cycling of soil carbon (C), nitrogen (N), and phosphorus (P) in the Binggou River Basin of the Eastern Qilian Mountains, focusing on three land types: forest, grassland, and cultivated land. The study demonstrates that land use changes influence the ecological stoichiometry of these elements, showing that conversion from forest or grassland to cultivated land reduces soil organic carbon (SOC) and nitrogen content, causing the land to shift from a "carbon sink" to a "carbon source." The study further highlights that soil nutrient availability varies across different land types, with phosphorus becoming a limiting nutrient in forest soils, particularly in autumn. The study suggests strategies such as forest and grassland restoration, enhanced carbon sequestration, and reduced use of agricultural fertilizers to promote soil health and nutrient balance, providing theoretical support for ecological restoration. This paper is highly worthy of publication. However, before formal acceptance, it is recommended that some revisions be made.

Main Comments
 Although the study includes a large sample size, the sampling period is limited to 2018-2019, and the data are primarily concentrated in a small geographical area. Therefore, the spatial diversity and seasonal variation of the region may not be fully captured. It is recommended to add a discussion on uncertainty.
 While the discussion section addresses the impact of climate factors on soil nutrients, it does not sufficiently explore the long-term effects of different climate change scenarios on soil nutrient dynamics, such as asymmetric seasonal or spatial warming, etc (doi: 10.3389/fpls.2023.1090204; doi: 10.3389/fevo.2021.757943). It is recommended to add relevant discussions.

***Response: Thank you for your suggestion. We have added a discussion on uncertainties in the discussion section (4.3) (Line 534-551), and the references have been cited as follows (doi:10.3389/fpls.2023.1090204; doi:10.3389/fevo.2021.757943). The specific content is as follows:***

*In this study, continuous sampling was conducted during the 2018-2019 growing seasons to compare the seasonal variation characteristics of soil carbon, nitrogen, and phosphorus in different land use types in the Beigouhe watershed on the northern slope of the eastern Qilian Mountains. This research provides an initial understanding of the impact of land use type changes and seasonal climate fluctuations on soil carbon, nitrogen, and phosphorus. However, the sampling was limited to the 2018-2019 growing seasons and a small part of the Beigouhe watershed, with a short time series and limited spatial coverage. Additionally, due to seasonal frozen soil, the absence of samples from non-growing seasons may have hindered capturing the full spatial diversity and seasonal changes of soil carbon, nitrogen, and phosphorus in the region. Meanwhile, this study attempts to analyze the impact of seasonal climate fluctuations (temperature and precipitation changes) on the stability of soil carbon, nitrogen, and phosphorus during the growing season (May-October), but due to the lack of non-growing season data and soil samples from larger spatial scales, the effects of asymmetric seasonal or spatial warming on land use types and soil nutrient stability could not be fully discussed (Jiangwei Wang, et al., 2021; Fusong Han, et al., 2023). Therefore, future work will focus on increasing soil sampling in non-growing seasons and expanding the spatial coverage of soil samples to further strengthen the research on the spatial diversity and seasonal variations of soil carbon, nitrogen, and phosphorus in the Beigouhe watershed.*

Minor Comments

***Response:*** *Thank you for your kind comments and suggestions.We have carefully revised the manuscript based on your feedback and sincerely hope that the revised version meets your expectations.*
1. Line 456: "conficts of interest" should be corrected to "conflicts of interest."

***Response 1:*** *Thank you very much for your valuable suggestions. We have corrected "conficts of interest" to "conflicts of interest."****(Line 610)***

2. Line 456: "exits" should be corrected to "exists."

***Response 2:*** *Thank you very much for your valuable suggestions. We have corrected "exits" to "exists."*
3. "microbial biomass phosphorus" should be revised to "microbial biomasses phosphorus."

***Response 3:*** *Thank you very much for your valuable suggestions. We have corrected "microbial biomass phosphorus" to "microbial biomasses phosphorus."*
4. Figure 2 could benefit from using monthly data to match the other figures.

***Response 4:*** *Thank you very much for your valuable suggestions. We have modified the data in the figure to monthly data.*
5. Figure 4 lacks an x-axis label and should be corrected.

***Response 5:*** *Thank you very much for your valuable suggestions. We have added the missing x-axis label to Figure 4 and have redrawn the figure accordingly.*
6. "This study analyzes" should be changed to "This study analyzed."

***Response 6:*** *Thank you very much for your valuable suggestions. We have corrected "This study analyzes" to "This study analyzed."*
7. Line 287: "The results show" should be changed to "The results showed."

***Response 7:*** *Thank you very much for your valuable suggestions. We have corrected "The results show" to "The results showed."*
8. The sentence "From 2018 to 2019, the average soil organic carbon (SOC) content in forest land (48.82 g/kg) was higher than in grassland and cultivated land" should be revised to: "From 2018 to 2019, the average soil organic carbon (SOC) content in forest land (48.82 g/kg) was found to be higher than that in grassland and cultivated land" to improve clarity.

***Response 8:*** *Thank you very much for your valuable suggestions. We have corrected "From 2018 to 2019, the average soil organic carbon (SOC) content in forest land (48.82 g/kg) was higher than in grassland and cultivated land" to "From 2018 to 2019, the average soil organic carbon (SOC) content in forest land (48.82 g/kg) was found to be higher than that in grassland and cultivated land."*
9. The color scheme in Figure 6 appears somewhat jarring and may need adjustment.

***Response 9: Thank you very much for your valuable suggestions. We have adjusted the color scheme in Figure 6 and redrawn the figure accordingly.***
10. The references section has some formatting issues, such as incorrect indentation (e.g., line 474).

***Response 10: Thank you very much for your valuable suggestions. We have revised the format of the references as required.***

Reviewer #2: Impacts of land use on soil carbon, nitrogen, and phosphorus cycling in the eastern Qilian mountains. This manuscript explores the spatial and temporal patterns in forest, grassland, and cultivated land and presents some interesting results. However, further modifications and improvements are necessary to enhance its clarity and rigor.
Regarding theme of the manuscript, which focuses on different land use, the relationship between forest, grassland and cultivated land remains unclear. Line 95-96 mentions a series of ecological restoration projects have been implemented in Qilian Mountains since 2000. What about the three samples? According to Line 123-124 and Line 215-216, the grassland and woodland have been converted into cultivated land. There are some contradiction and confusion for me. Therefore, the authors should provide to clarify the relationship between these samples.

***Response: Thank you for your questions and suggestions. We acknowledge that the expression of this issue was indeed unclear. Based on your suggestion, we have made revisions (Line 105-113).***

*In recent decades, global climate change has had a significant impact on the Qilian Mountain ecosystem. Additionally, the expansion of cultivated land and the reclamation of forests and grasslands by local residents have further degraded the ecosystem. Although China has implemented a series of ecological restoration projects since 2000, including natural forest protection, the construction of ecological public welfare forests, and the return of farmland to forest (or grassland) to protect and restore the degraded ecosystem (Wang et al., 2019), some of the reclaimed farmland has yet to be converted back to grassland or forest. Changes in land use types have altered ecosystem services and impacted the processes of soil nutrient retention and cycling.*
Furthermore, soil carbon, nitrogen and phosphorus cycling typically involve various processes, but this manuscript only considers soil organic carbon, nitrogen and phosphorus. Therefore, the title of the manuscript may need further consideration.

***Response:*** *Thank you for your suggestions. After careful consideration and in response to your feedback, we have revised the title of the paper.*

The title "Impacts of Land Use on Soil Carbon, Nitrogen, and Phosphorus Cycling in the Eastern Qilian Mountains" has been changed to "Impacts of Land Use on Soil Carbon, Nitrogen, and Phosphorus in the Eastern Qilian Mountains."

L14. The terms “forest” and “woodland” are used interchangeably in the manuscript. For consistency, the authors should choose one term and use it throughout the document.

***Response:*** *Thank you for your suggestion. We have made the revision and have standardized the use of "forest land" throughout the manuscript.*

L27. The statement that lower C:N and C:P ratios weaken soil microorganisms, hindering plant nutrient absorption and inhibiting root growth, is not supported by data. Therefore, the authors should revise the statement or cite references to be more accurate.

***Response:*** *Thank you for your suggestion. It is true that we did not test soil microorganisms, and that conclusion is a combination of existing research results (Du et al., 2020). , which was inferred from the C:N and C:P characteristics of the arable soils in this paper. We removed this sentence based on insufficient data to support this conclusion.*

Du, E., Terrer, C., Pellegrini, A.F.A. et al. Global patterns of terrestrial nitrogen and phosphorus limitation. Nat. Geosci. 13, 221–226 (2020). <https://doi.org/10.1038/s41561-019-0530-4>

L31. The conclusion that phosphorus becomes the limiting nutrient for forest growth in autumn cannot be directly inferred from the data presented. The authors should provide additional evidence or revise this statement to be more accurate.

***Response:*** *Thank you for your questions and suggestions. We have revised this paragraph of the summary as a whole*

L152, L179, L194. These statements mention the soil particle size of soil for the measurement of TN and TP, but there is a contradiction between 0.25 mm and 100-mesh sieve.

***Response:*** *Thank you for your questions and suggestions. Soil samples were air-dried for a fortnight in a cool, ventilated environment. Plant debris and gravel were removed from the soil samples prior to soil splitting. The air-dried soil samples were ground and passed through a 100 mesh sieve to remove coarse fragments and debris for the determination of soil organic carbon (SOC), soil total nitrogen (TN), and soil total phosphorus (TP).*

L192, L207. Equipment used in the manuscript should be marked with the model, manufacturer and country.

***Response:*** *Thank you for your questions and suggestions. We have revised the original text as follows:Soil sample measurements were carried out using the Smartchem 200 Discrete Auto Analyzer, branded AMS Alliance, Italy.*

L209-L210. Significance test for different land use, seasonal and so on are missing.

***Response:****Thank you for your suggestions. We have added the method for testing the significance level and conducted tests on the differences in carbon, nitrogen, and phosphorus content across different land use types, as well as the significance of seasonal variations.*

L274-275, L284-286, L339-342. These sentences, which serve as argument, should be included in the discussion section of the manuscript for better organization and clarity.

***Response:*** *Thank you for your questions and suggestions. We have incorporated these three sentences into the discussion section to better support our research, respectively in the subsections.*

L308-309, L330-331, L336-338. The sentence should be convincing evidence, based on the references or the data, to support these statements.

***Response: Thank you for your suggestion, we have carefully reviewed these sentences and have deleted and modified them as follows, taking into account that they do not explain the data in the text very well:***

L308-309：

***Response: Thanks to your suggestion. Based on the fact that during our survey of the study area we found that the grassland was grazed and that nitrogen fertiliser was used on the arable land, the following adjustments were made to the paragraph where L308-309 is located:***

*During the investigation of the study area, it was found that grazing existed in the grassland, and nitrogen fertiliser would be used in the arable farming process, which increased the input of nitrogen, but the carbon sequestration capacity of the grassland and the arable land was weaker (Fig. 3), and the consumption of nitrogen was limited by carbon, which couldn't be absorbed by the plants sufficiently, and it was accumulated in the soil, which resulted in the TN content of the soil of the grassland was higher than that of its month of July in summer, and the TN content of the soil of the arable land decreased, but to a lesser extent, in July (Fig. 3). month was reduced, but the reduction was small (Figure 3). On the other hand, the phosphorus content of grassland and arable soils decreased significantly in summer, especially in July, resulting in higher soil N:P ratios in summer.*

L330-331:

***Response: Thanks to your suggestion, we have amended the above sentence as follows：***

*Plant and animal residues mainly accumulate on the soil surface, and soil microorganisms are more active in the shallow layer with strong decomposition and phosphorus mineralisation capacity (Wang et al., 2021; Carrasco-Espinosa et al., 2024), resulting in the soil SOC and TN contents being the largest in the surface layer and decreasing rapidly with the increase of the soil layer, whereas the soil TP showed relatively stable characteristics, with insignificant changes with the increase of soil layers. As a result, the C:N to C:P ratio in the top soil layer (0-30 cm) gradually decreased.*

L336-338：

***Response:*** *Thanks to your suggestion, we have amended the above sentence as follows：*

*As previously described, with the increase in soil depth, the input of organic matter gradually decreases, microbial activity reduces, and decomposition weakens. The contents of soil SOC and TN gradually decrease with the increase in soil layers (with no obvious changes in cropland). However, the TP content in different soil layers of grassland and cropland is relatively stable, showing no obvious changes with the increase in soil layers. Although the TP content in forestland soil also shows a decreasing trend with the increase in soil layers, the rate of its decrease is lower than that of SOC and TN. This should be the main reason leading to the maximum C:P and N:P ratios in the surface soil of forestland, and the C:P and N:P ratios in the 0–30 cm soil layer of grassland soil being both greater than those in the 30–60 cm soil layer.*

L380. Climate change refers to changes in the climate over a long period of time. The data of manuscript just only involves from 2018 to 2019, then how to get the conclusion “climate change has the most significant impact on soil organic matter content in woodlands, followed by grasslands and cultivated lands”?

***Response:****Thank you for your questions and suggestions. This study only analyzes the impact of temperature and precipitation fluctuations during the growing seasons of 2018 and 2019 on soil carbon, nitrogen, and phosphorus. Therefore, the term "climate change" is not appropriate. We have made the following revision: "climate change" has been changed to "fluctuations in temperature and precipitation during the growing season."*

L382-383. In the manuscript, cultivated land through tillage, disrupts soil aggregates and increases ventilation lead to a weakened carbon sequestration capacity and an imbalanced stoichiometric ratio. How to get the result? Isn’t the lack of litter in cultivated land as a more important reason for the pattern of soil organic carbon?

***Response:*** *Thank you very much for your questions and suggestions. The point "while cultivated land, through tillage, disrupts soil aggregates and increases ventilation" is based on the findings of Li et al. (2012) and Wu et al. (2011). As you mentioned, the primary reason for the low soil organic carbon content in cultivated land is the lack of litter. Therefore, we have revised the original expression as follows:*

*The carbon sequestration capacity of cultivated land is relatively weak, mainly due to the lack of sufficient litter. Secondly, the destruction of soil aggregates and the enhancement of soil ventilation capacity also weaken the carbon sequestration capacity of soil(Li et al., 2012; Wu et al., 2011).*

L419. Please provide the description of table1. How to get the parameter of ecostoichiometric ratio?

***Response:****Thank you for your questions and suggestions. We have added an explanation for Table 1.*

*Table 1 shows the average values of soil carbon, nitrogen, phosphorus, and stoichiometric ratios in the 0-60 cm soil layer from May to October for cultivated land, forest land, and grassland. It is evident that there are significant differences in soil nutrient content and stoichiometric ratios across different land use types.*

The conclusion section is a summary of the research results, as well as an in-depth analysis of the research achievements and a prospect for future studies. It consolidates and refines these facts based on the research outcomes, while maintaining rigor by avoiding over-extrapolation (L434, L443, L446). The conclusion requires revisiting and reorganization.

***Response:****Thank you for your suggestions. We have reorganized the conclusions. The details are as follows:*

*This paper analysed the spatial and temporal changes of soil carbon, nitrogen and phosphorus contents and their ecological stoichiometric ratios in woodland, grassland and cropland soils in the Binggou River Basin in the eastern section of the Qilian Mountains based on continuous field sampling data. The effects of land use changes and seasonal climate fluctuations on soil carbon, nitrogen and phosphorus were discussed. The following main conclusions were obtained:*

*The SOC content of woodland soil was significantly higher than that of grassland and cropland, and the conversion of woodland and grassland into cropland decreased the soil SOC content by 16.04 g/kg and 4.8 g/kg, respectively. This decreased the soil carbon sequestration capacity significantly and increased the risk of soil carbon emission. Thus, in the future ecological restoration process, efforts to return ploughland to forest and grassland should be further increased to enhance the ecosystem service function of the Qilian Mountain area. The TP content of forest soil was significantly lower than that of grassland and cropland, and the difference in TN content was very small, resulting in significantly higher C:P and N:P ratios in forest soil than in grassland and cropland. Especially in autumn, the C:P and N:P ratios in forest soil were much higher than those in other seasons. Excessive C:P reduced the effectiveness of soil phosphorus. The weaker carbon sequestration capacity of arable land resulted in significantly lower C:N (10.13) and C:P (47.38) ratios than those of woodland and grassland. Lower C:N and C:P ratios weakened soil microbial activity, hindered nutrient uptake by plants, and inhibited root growth. In terms of seasonal changes, the SOC content of the three land use types showed the characteristic of “low in summer and high in spring and autumn”, while the TN content and C:N ratio of grassland soil were higher in summer and lower in spring and autumn, and the autumn TN content showed seasonal fluctuations contrary to those of woodland. In the soil vertical profile, the contents of SOC, TN, and TP in forest land showed a significant decreasing trend with the increase of the soil layer, especially in the 0-30 cm soil layer. The characteristics of changes in soil SOC and TN in the vertical profile of grassland soil were similar to those of woodland, but the maximum value of soil TP content appeared in the 50-60 soil layer. The three kinds of nutrients in arable soils showed an ”S” type of change in the vertical direction, and the reduction of tillage, ploughing, and the use of chemical fertilisers changed the distribution of soil nutrients in the vertical profile of the soils. In the future ecological restoration process, attention should be paid to the problem of low TP content and high C:P in autumn woodland. For arable land that still existed, measures such as returning straw to the field and applying organic fertilisers were taken to increase the input of organic matter, enhance the carbon sequestration capacity of arable land soils, improve soil fertility, prevent soil degradation, and promote plant growth.*

*Based on the existence of seasonal permafrost in the study area, this paper conducted continuous sampling from May to October in the plant growing season of 2018-2019, carried out a comparative study of soil carbon, nitrogen, and phosphorus characteristics of different land-use types in the Binggou River Basin on the northern slope of the eastern section of the Qilian Mountains, and preliminarily grasped the laws of land-use type changes and seasonal climatic fluctuations on soil carbon, nitrogen, and phosphorus. However, since the sample collection was limited to the plant growing season and localised areas in the Binggou River Basin, with a short time series, small sampling area, and lack of sampling data from non-growing seasons, it might not fully capture the spatial diversity and seasonal variation characteristics of regional soil carbon, nitrogen, and phosphorus. It was not possible to discuss the effects of asymmetric seasonal or spatial warming on land use types and soil nutrient stability more fully. Therefore, in future work, the collection of soil samples in the non-growing season should be increased, the spatial range of soil sample collection should be expanded, and the time series of sample collection should be lengthened to further study the spatial and temporal variation of soil carbon, nitrogen, and phosphorus in the Binggou River Basin in depth, as well as its driving mechanism.*

Reviewer #3: Taking the Binggou River Basin (north slope of the eastern Qilian Mountains), as study area, a sensitive area to global change and play a crucial role in maintaining regional ecological security as a national nature reserve. I had comprehensively and deeply reviewed the manuscript, which was entitled: The Impacts of Land Use on Soil Carbon, Nitrogen, and Phosphorus Cycling in the Eastern Qilian Mountains. This is a topic of some interest to researchers in related fields, but the paper needs a lot of improvement before it can be accepted. My detailed comments are as follows:

1. “Soil Carbon, Nitrogen, and Phosphorus Cycling”, How is it defined? How is this measured in your experimental design? The title of the manuscript does not correspond well with the research content.

***Response:*** *Thank you for your suggestions. We have made appropriate modifications to the manuscript title to ensure it aligns closely with the content of the study.*

*The title has been revised to: "Impacts of Land Use on Soil Carbon, Nitrogen, and Phosphorus in the Eastern Qilian Mountains."*

2. In terms of experimental design, only one plot is set for each land use type, and there is no duplication in land use. Can it support your research conclusions.

***Response:*** *Thank you for your questions and suggestions. Regarding the experimental design, indeed, only one plot was set up for each land use type, and within each plot, five subplots were established for repeated sampling to ensure representativeness. The measured data should support the conclusions of this study. However, based on your suggestion, we will consider expanding the sampling scope in future work to enhance the representativeness of the data. Thank you again for your valuable feedback.*

3. Do the three sampling plots of different types of land use have the same/similar conditions in terms of terrain, light, soil, etc.?

***Response:*** *Thank you for your questions and suggestions. In order to ensure comparability between different land use types, the parcels we have selected are in. They have similar conditions in terms of topography, light and soil.*

4. In terms of data analysis, ANOVA and significance test are lacking.

***Response:*** *Thank you for your suggestion. We have added analysis of variance (ANOVA) and significance level tests to more clearly quantify the differences in soil nutrients across different land use types and the seasonal fluctuation characteristics.*

5. The innovation of the research is not clearly expressed.

***Response: Thanks to your suggestion, we have added the innovation and importance of this paper in the preface section (Line 123-128). The details are as follows:***

*The innovation and significance of this paper are mainly reflected in the following two aspects: First, a comparative study was carried out on the changes of soil carbon, nitrogen and phosphorus in different land use types in the Qilian Mountains, and the impact of land use type transformation on soil carbon sequeeration capacity and soil nutrient balance was clarified. Second, the research results have certain guiding significance for ecological restoration in Qilian Mountains.*
