# Supplementary material for: Impacts of land use on soil carbon, nitrogen, and phosphorus in the Eastern Qilian Mountains
Source: PLoS One. 2025 Jul 14;20(7):e0326316. doi: 10.1371/journal.pone.0326316 (PMC12258590; doi:10.1371/journal.pone.0326316)
Supplement: Attachment — Submitted filename: Response to Reviewers1.doc [file pone.0326316.s005.doc]

***Dear Editor and Reviewers:***

***We are grateful for your consideration of our manuscript entitled "Impacts of Land Use on Soil Carbon, Nitrogen, and Phosphorus Cycling in the Eastern Qilian Mountains" [PONE-D-24-59609R1], and we also very much appreciate your constructive comments and useful suggestions, which have enabled us to improve the manuscript. All the comments we received on this study have been considered, and we present our reply to each of them separately. We hope the revised manuscript will satisfy you.The following is an illustration of the different coloured revisions in the manuscript with markings: blue with strikethrough are markings removed from the original text, yellow highlights are reworked additions.***

Reviewer #2: The revised manuscript shows substantial improvement and now ready for minor revisions before formal acceptance. Please address the following points:

1.L182, L196: The capitalization of the initial letters in the title should be consistent throughout. Please standardize this.

***Response: Thank you for your suggestion. We have standardised the case of initial letters in the title and the title has been amended as follows:***

***Impacts of land use on soil carbon, nitrogen, and phosphorus in the Eastern Qilian Mountains***

2.L 250, L251, L266, L285, Fig.4 and Table 1: It is recommended to include variability indicators (±SD or ±SE) for the key indicators in the paper.

***Response: Thank you for your suggestion. We have added variability indicators (±SD) at these locations, of which the modifications to Table 1 are shown below:***

*Table.1 Soil carbon, nitrogen and phosphorus contents and ecological stoichiometric ratios of different land use types. Values are average±standard error.*

|  | *SOC(g/kg)* | *TN(g/kg)* | *TP(g/kg)* | *C/N* | *C/P* | *N/P* |
| --- | --- | --- | --- | --- | --- | --- |
| *Forestland* | *48.82±7.59* | *3.52±0.77* | *0.46±0.09* | *13.87±6.11* | *106.13±48.82* | *7.65±3.22* |
| *Grassland* | *38.32±5.87* | *2.85±0.44* | *0.58±0.04* | *13.45±6.54* | *66.07±11.80* | *4.91±0.98* |
| *Farmland* | *30.8±5.94* | *3.04±0.25* | *0.65±0.07* | *10.13±1.98* | *47.38±8.32* | *4.67±0.79* |

3.“p” in the manuscript: The letter “p” should be represented in lowercase and italicized.

***Response: Thank you for your suggestion. We have represented all letter “p” in the manuscript in lowercase and italicised.***

4.L447. “soil SOC” is the abbreviation of “soil soil organic carbon”, please revise it.

***Response: Thank you for your suggestion. To ensure the consistency of the article, we will uniformly change "soil SOC" to "SOC" throughout the text.***

5.L494. TN has already been defined in the previous text, so there is no need to define it again.

***Response: Thank you for your suggestion. As you pointed out, the definition of TN has already been presented earlier in this paper. Therefore, we have removed the duplicate definition here.***

6.Please modify the references according to the format requirements.

***Response: Thank you for your suggestion. We have changed the format of the references according to the journal requirements***

Reviewer #3: This version of the manuscript responds to the comments of the three reviewers one by one, and the quality of the manuscript has been greatly improved, but more specific and comprehensive information needs to be provided in some details through minor revision. My main suggestions are as follows:

1.Firstly, in the section “2.1 Description of the Study Area”, It is suggested to supplement the land use and cover changes of the whole study area over the past 10 years, especially the plant composition and community structure characteristics of each sampling site and the tillage of agricultural land.

***Response: Thank you for your suggestion. We have extracted the data on land use and cover change in the study area over the past ten years, and added the calculation results to the introduction section. In addition, the characteristics of the plant composition and community structure at each sampling point, as well as the cultivation situation of the agricultural land, have been added to the introduction of the study area, which is specifically as follows:***

*Line109-Line112: From 2009 to 2019, the farmland area increased by 1.0278km^2^; the forestland area increased by 4.4199km^2^; the grassland area decreased by 6.246km^2^; the water body area decreased by 0.4761km^2^; the unused land area increased by 1.2744km^2^; and the Build-upland area remained unchanged.*

*Line154-Line165: In the Picea crassifolia forest, the canopy density of the arbor layer is relatively high, which leads to insufficient light beneath the forest. Consequently, shrubs exhibit poor growth and development, resulting in relatively low species diversity. This forest community is mainly composed of deciduous shrubs, such as Potentilla fruticosa and Potentilla glabra from the Rosaceae family. The herb layer predominantly comprises typical alpine meadow plants, including Carex sp., Polygonum viviparum, and Pedicularis sp. The bryophyte layer is well - developed, primarily consisting of Abietinella abietina, Hypnum cupressiforme, Mnium cuspidatum, etc. The grassland community features a simple structure, mainly composed of species like Stipa spp., Agropyron cristatum, and Oxytropis spp. Farmland in the Ice Gully River Basin was mainly located along the river and was the result of the development of riparian forestland and grassland. The arable land at the sampling site had been cultivated for 32 years and was mainly planted with wheat and potatoes.*

2.Secondly, It is proposed to supplement the hydrological connectivity status of the Binggou River Basin, including the interaction of surface runoff in its adjacent catchments.

***Response: Thank you for your suggestion. We have added the following to the introduction to the study area:***

*Line136-Line138: The Binggou River watershed consists of two tributaries: the Binggou River and the Nancha River, and the Binggou River watershed is 45 km long with a watershed area of 335 km^2^.*

3.Thirdly, Please state what damage has been done to the ecological environment in the study area and to what extent.

***Response: Thank you for your suggestion.We have found that the reclamation of forests and grasslands by local residents further damaged the ecosystem. Therefore, by compared the local land use data of the past ten years, the following results were obtained and added to the introduction part.***

*Line109-Line112: From 2009 to 2019, the farmland area increased by 1.0278 km^2^; the forestland area increased by 4.4199 km^2^; the grassland area decreased by 6.246 km^2^; the water body area decreased by 0.4761 km^2^; the unused land area increased by 1.2744 km^2^; and the Build-upland area remained unchanged.*

Others, (1) There is room for improvement in some series of results analysis, for example, in Fig 7, Columns filled with patterns and colors only indicate the meaning of grassland, farmland and forestland are not marked.

***Response: Thank you for your suggestion. We have modified Figure 7 by adding the legend below:***

(2) In terms of the content arrangement of this manuscript, the results and the discussion are separate. It is suggested that the discussion expressions quoting references in the results should be included in the discussion chapter.

***Response: Thank you for your suggestion. The following sentences have been placed in the Discussion section in previous drafts:***

*Line 506-507: The lower the C:N ratio, the faster nitrogen is released into the soil for crop use (Watson et al. 2002).*

*Line 517-519: The soil C:P ratio is an important indicator of phosphorus mineralization capacity and a potential measure of soil organic matter's ability to release or absorb phosphorus from the environment (Zhang et al. 2017).*

*Line 495-500: This indicates that the surface soil exhibits a strong nitrogen mineralization capacity. As decomposition progresses, easily degradable material is consumed, and nitrogen is immobilized in microbial biomass and decay products. This leaves behind more recalcitrant material, which decomposes at slower rates (Post et al., 1985).*

***Response: In addition, we have added some parts of the following results to the discussion.***

*Line 412-413 were added to Line 531-533: The C:N ratio of farmland fluctuated around 10.18, and the C:N ratio of soil surface organic matter under conventional tillage ranged from 8 to 15, with an average of 10 to 12 (Huang, 2000). In this paper, the soil C:N ratio of arable land was 10.13 ± 1.98 g/kg, which was lower than that of grassland and forestland.*

*Line 419-421 were added to Line 543-549: Plant and animal residues mainly accumulate on the soil surface, and soil microorganisms are more active in the shallow layer with strong decomposition and phosphorus mineralisation capacity (Wang et al., 2021; Carrasco-Espinosa et al., 2024). This resulted in higher TP levels in the surface soils of forestland, grassland and arable land than in the middle and deeper layers.*

(3) The evidence (references) cited in the discussion section should have similar research premises to this study, please check.

***Response: Thank you for your suggestion. We have updated the references and removed those that are not relevant to this study.***

(4) When it comes to the significance level of the impact/difference, it is recommended to indicate a probability value, such as p < xxx.

***Response: Thank you for your suggestion. We added probability values (P<0.05) at the places where significance tests occurred.***
